# Predicting the Net Energy Partition Patterns of Growing Pigs Based on Different Nutrients

**DOI:** 10.3390/ani15162464

**Published:** 2025-08-21

**Authors:** Wenjun Gao, Zhengcheng Zeng, Huangwei Shi, Lu Wang, Shijie Liu, Xinwei Dong, Tenghao Wang, Changhua Lai, Shuai Zhang

**Affiliations:** 1 State Key Laboratory of Animal Nutrition and Feeding, College of Animal Science and Technology, China Agricultural University, Beijing 100193, China; wjgao1119@163.com (W.G.); zengzhengcheng@cau.edu.cn (Z.Z.); shw1161402802@163.com (H.S.); wanglucau@163.com (L.W.); 2 Chongqing Sinopig High-Tech Group Co., Ltd., Chongqing 402460, China; liushijie@guozhu.net (S.L.); dongxinwei@guozhu.net (X.D.); 3 Zhejiang Qinglian Food Co., Ltd., Jiaxing 314399, China; wangth85@126.com

**Keywords:** energy-supplying nutrients, ingredient, net energy partition patterns, prediction equation, growing pigs

## Abstract

This study aimed to determine the net energy (NE) values of common energy-supplying nutrients, including starch, protein, and fat, to investigate their influence on energetic efficiency and NE partition patterns in growing pigs, and to develop prediction equations for the protein deposition (PD) and lipid deposition (LD) based on nutrient characteristics of ingredients. Two experiments were conducted. In Experiment 1, 36 growing barrows (Duroc × Landrace × Yorkshire, initial body weight = 28.1 ± 0.8 kg) were randomly allotted to six treatments, with six replicated pigs per treatment. The diets were formulated as follows: a corn–soybean meal basal diet (T1), and five experimental diets containing of 27% corn starch (T2), 27% tapioca starch (T3), 27% pea starch (T4), 5% soybean oil (T5), and 11.8% casein (T6), respectively. In Experiment 2, PD and LD data of 47 ingredients were collected. Subsequently, the nutrient characteristics of ingredients were used as input variables, and PD and LD were used as output variables to establish the prediction equations. Results exhibited that pigs fed the T2, T3, and T4 diets showed increased digestibility of gross energy (GE) and organic matter (OM) compared to those fed the T1 diet (*p* < 0.01). For various kind of starches, a greater efficiency of using metabolizable energy (ME) for net energy not deposited as protein (PD-free NE, efficiency denoted as *kj*) was observed when pigs were fed the T2 or T3 diets compared to the T4 diet. Moreover, the *kj* of soybean oil was 11% and 27% greater than that of starch and casein, respectively, while casein demonstrated 46% and 39% greater efficiency of using ME for PD (efficiency denoted as *pj*) compared to starch and soybean oil, respectively. Finally, the best-fitted prediction equations for PD and LD were PD = 364.36 − 18.44 × GE + 29.10 × CP − 3.79 × EE − 21.37 × ADF (R^2^ = 0.96; RMSE = 105.15) and LD = −1503.50 + 21.58 × CP + 51.98 × EE + 26.30 × Starch + 26.81 × NDF − 23.87 × ADF (R^2^ = 0.98; RMSE = 172.85), respectively. In summary, there are considerable differences in energetic efficiency and NE partition patterns among various nutrients. In addition, PD and LD can be predicted through nutrient characteristics of ingredients, presenting an innovative approach and methodological framework for the precision nutrition of pigs.

## Simple Summary

The net energy (NE) system integrates the energy value of ingredients with the energy requirements of animals, facilitating a detailed classification of NE for growing pigs into protein deposition (PD) and lipid deposition (LD) using a factorial method. Considering the nutrient characteristics of ingredients have a substantial impact on the NE partition patterns, further research in this area is imperative. In this study, common nutrient monomers were employed to investigate their influence on energetic efficiency and NE partition patterns, while also developing prediction equations for PD and LD, considering the nutrient characteristics. Corn starch and tapioca starch exhibited greater energetic efficiencies in comparison to pea starch. Soybean oil was 11% and 27% more efficient than starch and casein in using metabolizable energy (ME) for net energy not deposited, while casein showed 46% and 39% higher efficiency than starch and soybean oil for PD. In addition, the best-fitted prediction equations for PD and LD were PD = 364.36 − 18.44 × GE + 29.10 × CP − 3.79 × EE − 21.37 × ADF (R^2^ = 0.96; RMSE = 105.15) and LD = −1503.50 + 21.58 × CP + 51.98 × EE + 26.30 × Starch + 26.81 × NDF − 23.87 × ADF (R^2^ = 0.98; RMSE = 172.85), respectively.

## 1. Introduction

Compared with the digestible energy (DE) and metabolizable energy (ME) systems, the net energy (NE) system offers a more accurate assessment of the energy requirements of animals and the energy value of ingredients [1]. However, the complexity of NE measurement limits the data acquisition to some extent.

To circumvent this limitation, the NE value can be predicted by multiplying the ME value by the efficiency of using ME for NE (k-value) [2]. The concept of the k-value is dynamic, as it is greatly influenced by the major energy-supplying nutrients in animal diets, such as carbohydrates, proteins, and fats. Starch, a key carbohydrate, demonstrates an average energy efficiency of 82% [3,4]. Additionally, different types of starch differ in chemical compositions, molecular structures, and physicochemical properties, thereby significantly influencing the k-value [5,6]. Under conditions of energy deficiency, proteins undergo oxidation and decomposition to generate energy; meanwhile, research has revealed that the k-value of soybean meal (a protein ingredient) stands at 60% [7,8,9]. The efficiency of fat hinges on the conversion process. Specifically, the k-value can reach up to 90% when stored as body fat, while it can decrease to 66% when oxidized for energy [7,10]. However, for specific energy-supplying nutrients (corn starch, tapioca starch, pea starch, soybean oil, and casein), no relevant research on their k-values has been carried out, which limits the comprehensive understanding of the energy value of these substances.

A previous study developed a multivariate weighted–nested regression model to clarify the NE partition patterns and further determine the k-value [11]. This model delineates NE into two components: protein deposition (PD) and net energy not deposited as protein (PD-free NE), and the efficiencies of these two components are represented as *pj* and *kj*, respectively. The PD-free NE is further differentiated into lipid deposition (LD) and ATP synthesis. This approach improves the understanding of NE partition patterns in animals and enables a more accurate assessment of the k-value. The majority of existing studies on predicting NE partition patterns are based on metabolic energy intake (MEI), such as estimating PD and LD through the difference between MEI and metabolizable energy for maintenance (ME_m_), as well as employing linear regression models [12,13,14,15]. However, it is well-known that nutrient sources exert a substantial and multifaceted influence on energy partition patterns. Therefore, integrating nutrient information, especially the information of nutrient characteristics of ingredients, into prediction equations can provide greater feasibility and practicality over MEI and ME_m_, which is currently still a research gap.

Based on this, the objective of this study was to determine the NE values and k-values of energy-supplying nutrients, including corn starch, tapioca starch, pea starch, soybean oil, and casein, to assess their effects on NE partition patterns when fed to growing pigs, and to develop prediction equations for PD and LD based on nutrient characteristics of different nutrients. This study could help explore an approach for achieving precision nutrition for pigs.

## 2. Materials and Methods

The animal trial in this study was conducted at FengNing Swine Research Unit of National Feed Engineering and Technology Research Center, Ministry of Agriculture and Rural Affairs (Hebei Province, China).

### 2.1. Experiment 1

#### 2.1.1. Experimental Design and Diets

Thirty-six growing barrows (Duroc × Landrace × Yorkshire, initial body weight = 28.1 ± 0.8 kg) were selected and randomly allotted to one of six dietary treatments with six replicated pigs per treatment. The diets were formulated as follows: a corn–soybean meal basal diet (T1) and 5 experimental diets containing of 27% corn starch (T2), 27% tapioca starch (T3), 27% pea starch (T4), 5% soybean oil (T5), and 11.8% casein (T6), respectively.

According to the experimental protocol of the difference method, the proportions of energy-supplying fractions in each diet were kept consistent, with a corn/soybean meal ratio of 3.41 [16]. Meanwhile, the total proportions of amino acids, minerals, and premixes were consistent across all diets (Table 1). All of the nutrient levels were adjusted to meet or exceed the nutrient requirements of growing pigs or adult sows (NRC, 2012) [8].

#### 2.1.2. Experimental Management and Sample Collection

All pigs were fed at a level of 1.92 MJ ME/kg BW^0.6^/d, and had free access to water during the whole animal trial. The animal trial included six periods, each consisting of a 7-day diet adaptation period followed by a 7-day HP measurement period. From days 0 to 7, pigs were individually housed in stainless steel digestive–metabolic cages (1.40 × 0.70 × 0.60 m^3^). On day 8, pigs were transferred to the chambers for HP measurement. On day 13, pigs were fasted, and fasting heat production (FHP) was measured from 22:30 on day 13 to 06:30 on day 14. The temperature of the respiratory calorimetry chamber was controlled at 22 ± 1 °C and relative humidity at 70% ± 5%. Pigs were weighed at the beginning of each period on days 1, 7, 13, and 14 to calculate the amount of feed supplied.

Feces and urine from each pig were collected twice daily during the HP measurement period. At the end of each collection period, the 5 d fecal samples were mixed, weighed, dried in an oven at 65 °C for 72 h, and ground through a 1 mm screen before chemical analysis. At the same time, the 5 d urine samples were collected into plastic buckets containing 50 mL of 6N HCl for nitrogen fixation and mixed well, and 10% of the total urine samples were stored at −20 °C for subsequent analysis. The respiratory calorimetry device was utilized to record indoor gas data and outdoor environmental parameters at a frequency of every 5 min to calculate the HP and FHP of the test pigs.

#### 2.1.3. Chemical Analysis

The diets and fecal samples were analyzed for dry matter (DM; procedure 930.15) and ash (procedure 942.05) using the Official Methods of Analysis of AOAC International (2007) [17]. The diets and fecal samples were analyzed for ether extract (EE) using the method of [18]. Neutral detergent fiber (NDF) and acid detergent fiber (ADF) were determined with fiber analyzer equipment (Fiber Analyzer, Ankom Technology, Macedon, NY, USA), employing a procedure of Van Soest et al. [19]. All diets, urine (4 mL), and fecal samples were analyzed for crude protein (CP; procedure 984.13) according to the Official Methods of Analysis of AOAC International (2007) [17]. The gross energy (GE) in the nutrients, diets, urine (4 mL), and fecal samples were measured using an adiabatic oxygen bomb calorimeter (Parr 6300 Calorimeter, Moline, IL, USA).

#### 2.1.4. Calculations

The content of organic matter (OM) was determined by subtracting the ash content from the DM content. The apparent total tract digestibility (ATTD) of energy and nutrients were calculated as follows [16]:

ATTD = (ND − NF)/ND × 100%(1)

where ND is the total intake of energy or nutrients for an individual pig and NF is the total fecal output of energy or nutrients for an individual pig.

The nitrogen retention and net protein availability were calculated as follows:

Nitrogen retention = N intake − fecal N − urinary N(2)

Net protein availability = (N intake − fecal N − urinary N)/N intake × 100%(3)

where nitrogen retention is an absolute metric that directly quantifies daily protein deposition and correlates with growth performance, and net protein availability is a ratio-based efficiency metric reflecting nitrogen efficiency.

The DE value and ME value were calculated using the following equations [16]:

DE_i_ = GE_i_ − GE_f_ (4)

ME_i_ = GE_i_ − GE_f_ − GE_u_ − CH_4_E (5)

where the subscript i indicates the diets in the experiment, and GE_i_ is the total GE intake from diets (MJ/d), GE_f_ is the total fecal output of energy (MJ/d), GE_u_ is the total urinary output of energy (MJ/d), and CH_4_E is methane energy (MJ/d).

The content of heat increment (HI) was determined by subtracting the FHP from the THP. The THP and FHP were calculated as follows [20]:

HP = 16.18 × O_2_ + 5.02 × CO_2_ − 2.17 × CH_4_ − 5.99 × urinary N (6)

HI = THP – FHP(7)

From days 9 to 13 of each experimental period, measurements of O_2_, CO_2_, and CH_4_ concentrations were utilized to calculate THP at 5 min intervals. FHP was derived from the airflow data collected from 22:30 on day 13 to 06:30 on day 14, as described in Wang et al. [21].

The NE value, retention energy (RE), PD-free NE, and energetic efficiencies were calculated using the following equations [2,11,16]:

NE_i_ = ME_i_ – HI (8)

RE_i_ = ME_i_ − THP (9)

PD_i_ = (N intake − fecal N − urinary N) × 6.25 × 23.86 (10)

PD-free NE_i_ = NE_i_ − PD_i_ (11)

LD_i_ = RE_i_ − PD_i_ (12)

where 6.25 represents the conversion factor from nitrogen to protein, and 23.86 signifies the energy provided by 1 g of protein. Table 2 shows the variables used in Equations (8)–(12), including their definitions and units.

Energy value_j_ = (energy value_test_ − energy value_basal_/r_0_ × r_1_)/r_2_ (13)

*dj* = DE_j_/GE_j_ (14)

*mj* = ME_j_/DE_j_ (15)

*pj* = PD_j_/ME_j_ (16)

*kj* = PD-free NE_j_/ME_j_ × (1 − *pj*) (17)

LD_j_ = *kj* × (1 − *pj*) × ME_j_ − K_BR_ × FHP + NE_PD_ × PD_j_ (18)

where the subscript j indicates the nutrients, which include corn starch, pea starch, tapioca starch, soybean oil, and casein. Table 3 shows the variables used in Equations (13)–(18), including their definitions and units.

**Figure 1:**
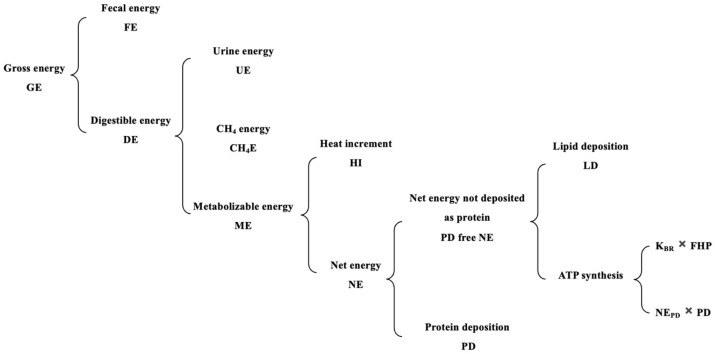
Energy utilization and partition in growing pigs based on multivariant weighted–nested regression model. GE, gross energy; FE, fecal energy; DE, digestible energy; UE, urine energy; CH_4_E, methane energy; ME, metabolizable energy; HI, heat increment; NE, net energy; PD-free NE, net energy not deposited as protein; PD, protein deposition; LD, lipid deposition; K_BR_, efficiency of using body reserves for ATP synthesis; THP, total heat production; NE_PD_, additional energy required for PD.

Figure 1 displays the energy partition patterns of growing pigs.

#### 2.1.5. Statistical Analysis

All data were tested for normality and homogeneity of variances with Q-Q plots using descriptive tests of SPSS (version 26.0, USA). Outliers were detected and excluded from subsequent analysis. The indices of digestibility and energy balance were analyzed with the one-way analysis of variance (ANOVA) procedure of SPSS. Multiple comparisons of the data were performed using Tukey’s test. Energetic efficiency and NE partition pattern data were nonlinearly fitted using the “Professional Modeling” program in JMP Pro 14.0 (SAS Institute, Carry, NC, USA). The model was y = x_1_ − x_2_ × a − x_3_ × b, where the variables y, x_1_, x_2_, and x_3_ represent LD, PD-free NE, THP, and PD, respectively; the parameter a represents K_BR_, which was set to an initial value of 1; the parameter b represents NE_PD_, which was set to an initial value of 0.5. The model parameters were solved by the Newton–Raphson iterative method, with the stopping limit set to 1000 and the stopping limit for target changes set to 10–15. In all analyses, significance was determined at *p* < 0.05, with a trend towards significance observed for 0.05 ≤ *p* < 0.10.

### 2.2. Experiment 2

#### 2.2.1. Data Sources

The experiment used data sourced from 47 ingredients, including 19 energy ingredients, 7 lipids, and 21 protein ingredients, which were previously evaluated in our laboratory. The data encompassed the nutrient content of each ingredient, as well as the corresponding PD and LD values measured in growing pigs. The nutrient content data of each ingredient, including GE, CP, EE, starch, NDF, ADF, ash, PD, and LD, were extracted and are listed in detail (Table 4). The PD and LD measurements of each ingredient contained six replicates with a corn–soybean meal diet, measured using the indirect calorimetry method on Duroc × Landrace × Yorkshire growing barrows at the same swine farm as in Experiment 1.

#### 2.2.2. Statistical Analysis

The data were first imported into Origin 2022 (V22, OriginLab Corporation, Northampton, MA, USA) for correlation analyses using the “Correlation Plots” package, adjusting XY variables, plotting labels, and generating heat maps. Multiple regression equations were fitted using the “Professional Modeling” program in JMP Pro 14.0 (SAS Institute, Carry, NC, USA). The input variables were nutrient characteristic contents and the output variables were PD and LD. Seventy percent of the data were used as the training set and the remainder as the validation set. The model qualities were stepwise, and the stopping rule was a *p*-value threshold of 0.05 for both entry and exclusion from the model. The R^2^, root mean square error (RMSE), Akaike Information Criterion (AIC), Bayesian Information Criterion (BIC), and validation R^2^ were recorded for each model. The model with the highest R^2^ and validation R^2^ values, and the lowest RMSE, AIC, and BIC values, was considered the optimal prediction model.

## 3. Results

### 3.1. Experiment 1

#### 3.1.1. Nutrient Digestibility and Nitrogen Balance of Experimental Diets

As shown in Table 5, the ATTD of GE and OM of growing pigs fed the T2, T3, and T4 diets were significantly greater than that of pigs fed the T1 diet (*p* < 0.01). Pigs fed the T5 diet exhibited greater ATTD of EE than in the other diets (*p* < 0.01). However, the ATTD of nutrients among the T2, T3, and T4 diets revealed no significant differences in growing pigs (*p* > 0.05).

Nitrogen intake increased with incremental levels of CP in the diets, exhibiting a significant difference (*p* < 0.01). Pigs fed the T2, T3, and T4 diets exhibited lower fecal nitrogen output compared to those receiving the T1, T5, and T6 diets (*p* < 0.01), whereas no significant difference was observed within the T2, T3, and T4 diets (*p* > 0.05). The T6 diet showed significantly higher urinary nitrogen output and lower net protein availability compared to the other diets (*p* < 0.01). Compared to the T1 or T5 diets, pigs fed the T2, T3, and T4 diets had lower nitrogen retention (*p* < 0.01), while no difference was exhibited in nitrogen retention within the T2, T3, and T4 diets (*p* > 0.05).

#### 3.1.2. Energy Balance of Experimental Diets

As shown in Table 6, pigs fed the T6 diet exhibited significantly greater PD and lower LD compared to those fed other diets (*p* < 0.01). In addition, the PD in the T2, T3, and T4 diets were lower than the T1 and T5 diets (*p* < 0.01), with no significant differences in PD and LD values observed among the various starch diets (*p* > 0.05).

The DE, ME, and NE values of the T5 diet were significantly greater than that of the T1 diet (*p* < 0.01). Furthermore, the ME and NE values of the T5 diet surpassed that of the T3 diet (*p* < 0.01), although no significant difference was observed in the DE value (*p* > 0.05). Moreover, the DE value of the T6 diet surpassed that of the T1 diet (*p* < 0.01). The ME/DE ratio of the T6 diet was significantly lower compared to that of pigs fed other diets (*p* < 0.01).

#### 3.1.3. Energy Values and Energetic Efficiencies of Nutrients

As shown in Table 7, the *pj* values of different starches were similar, with the value being 5%. The *kj* of corn starch and tapioca starch were comparable, with an average value of 87%, which was higher than that of pea starch. In addition, the *pj* of in casein was the greatest at 51%, whereas the *kj* of soybean oil was the most efficient at 95%. In the current study, it was estimated that the NE_PD_ was 0.35, indicating an efficiency of 0.35 for additional energy required for PD, and the K_BR_ was 0.96, indicating an efficiency of 0.96 in utilizing body reserves to supply energy during fasting.

Within different starches, the DE, ME, and NE values of tapioca starch were higher than those of corn starch and pea starch. Furthermore, within different nutrients, the DE, ME, and NE values of soybean oil were higher than the corresponding values of starch and casein. The NE values of corn starch, pea starch, tapioca starch, soybean oil, and casein on growing pigs in this experiment were 15.06, 14.37, 15.22, 34.03, and 16.22 MJ/kg DM, respectively.

### 3.2. Experiment 2

Correlation analysis and prediction equations for PD and LD based on nutrient characteristics of different ingredients.

**Figure 2:**
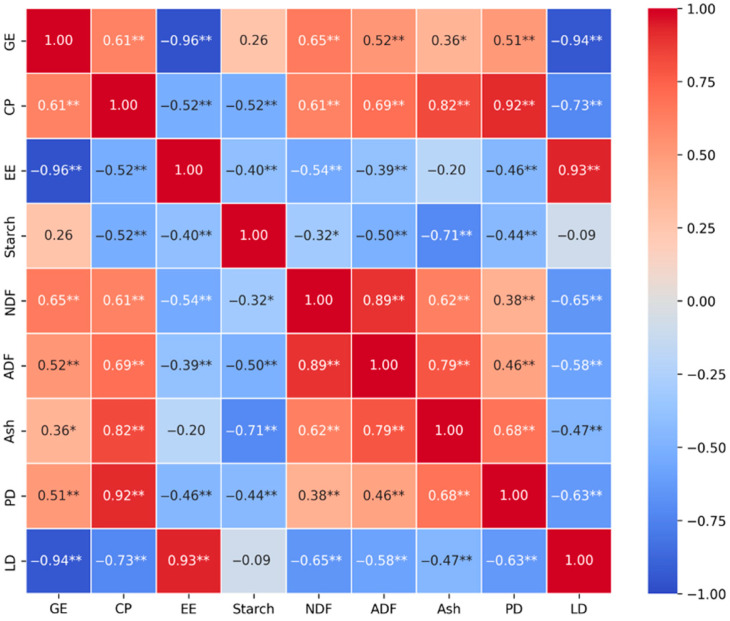
Correlation analysis between nutrient characteristics and protein deposition (PD) as well as lipid deposition (LD). The darker color indicates a greater correlation and the lighter color indicates a lower correlation. * represents 2 factors that were significantly correlated with *p* ≤ 0.05. ** represents 2 factors that were significantly correlated with *p* ≤ 0.01. PD, protein deposition; LD, lipid deposition; GE, gross energy; CP, crude protein; EE, ether extract; NDF, neutral detergent fiber; ADF, acid detergent fiber.

Figure 2 reveals significant correlations (*p* < 0.05) between the nutrient characteristics of ingredients and the corresponding net energy partition metrics PD and LD. As expected, the most substantial correlation was identified between PD and CP, with a coefficient (r) of 0.92. In the context of LD, the most substantial positive correlation is with EE, with an r value of 0.93, whereas the most notable negative correlation is with GE, yielding an r value of −0.94. However, the starch content showed no significant correlation with the net energy partition metrics.

As shown in Table 8, the CP and GE content of ingredients can be used as the single predictor to establish the univariate regression equations for PD and LD, respectively. The prediction equations derived were as follows: PD = −23.37.62 + 23.50 × CP (R^2^ = 0.87; RMSE = 177.36) and LD = 3106.14 − 141.09 × GE (R^2^ = 0.88; RMSE = 359.68). In the current study, the optimal prediction equation developed for PD was a quaternary equation comprising GE, CP, EE, and ADF, while that for LD was a quintuple prediction equation with factors including CP, EE, starch, NDF, and ADF.

## 4. Discussion

### 4.1. Nutrient Digestibility and Nitrogen Balance of Experimental Diets

Starch, soybean oil, and casein are known as high-quality nutritional nutrients for growing pigs, recognized for their rapid and efficient enzymatic breakdown in the digestive system [22,23,24,25,26]. In this study, it was found that the source of starch did not affect the ATTD of nutrients in growing pigs, likely due to their efficient degradation capabilities [6]. The addition of soybean oil significantly increased the ATTD of EE in the experimental diet, a result similar to that reported in a previous study [27], which was attributed to the greater digestibility of soybean oil compared to corn–soybean meal, starch, and casein in the diet.

Fecal nitrogen output primarily consists of undigested dietary nitrogen, endogenous losses, and microbial protein [28]. In this study, we observed a significant decrease in fecal nitrogen output from pigs fed the T2, T3, and T4 diets compared to those fed the T1, T5, and T6 diets. This reduction is primarily attributed to the lower nitrogen intake of these pigs. Noblet et al. [29] found that increasing the CP level in diets resulted in excess amino acids being metabolized, which in turn led to an increase in urinary nitrogen excretion. Zhang [30] furthered this by showing that urinary nitrogen output rose to 7.30 g/day with a 15.0% casein supplement in a rice starch diet in growing pigs. Our study indicated a higher output—8.34 g/day—when 11.8% casein was added to a corn–soybean oil diet. Collectively, these findings indicate that exceeding the CP requirement in animals leads to an increase in urinary nitrogen output. Pigs fed the high-protein diet (T6 diet) showed higher nitrogen retention compared to those fed other diets, and were also accompanied by higher PD. This is likely attributable to the fact that the amino acid profiles in the T6 diet are adequately abundant, particularly with respect to essential amino acids, including lysine, methionine, and threonine. However, pigs fed the T6 diet (Lys/ME ratio = 3.41 g/Mcal, exceeding the Chinese Swine Nutrient Requirements standard of 3.15 g/Mcal) exhibited 2.00% lower net protein availability compared to those receiving other diets (Lys/ME ratio = 2.41 g/Mcal) [31]. This reduction aligned with 4.66% higher urinary nitrogen excretion, likely attributable to the elevated Lys/ME ratio and excessive amino acids leading to increased nitrogen catabolism.

### 4.2. Energy Balance of Experimental Diets

In the current study, pigs fed the T6 diet exhibited greater PD, while those fed the T2, T3, T4, and T5 diets showed greater LD, indicating that the form of energy deposition in growing pigs is significantly influenced by the nutrient composition and level in the diet [32]. A linear relationship between the dietary CP level and PD was observed when MEI was maintained at a constant level [33]. Soybean oil can be directly utilized for fat synthesis with a high efficiency, while starch and casein show efficiencies of 74% and 53% for fat synthesis, respectively, which could be one explanation for the greater LD in growing pigs fed diets supplied with lipids [7]. But further studies controlling for key confounding factors across diets are needed to elucidate the specific mechanistic roles of PD and LD. There was no significant difference in the RE (PD + LD = RE) between the six treatment diets used in this study, suggesting that LD, calculated as RE minus PD, was negatively correlated with PD [34]. This correlation is consistent with the observed differences in PD and LD among diets, where the T6 diet showed higher PD with lower LD, while the T2, T3, T4, and T5 diets exhibited higher LD with lower PD.

The energetic efficiency analysis revealed that the T6 diet displayed a lower ME/DE ratio when compared to the other diets, whereas the ME/DE ratio showed a correlation with the increased urinary nitrogen output [35]. The result also confirms the results of Noblet et al. [2], namely, a negative correlation between dietary CP levels and the ME/DE ratio of the diets, indicating that a high Lys/ME ratio (3.41 g/Mcal) in the diet results in the waste of excess amino acids as urinary nitrogen, thereby reducing the ME/DE ratio in casein diet.

### 4.3. Energy Values and Energetic Efficiencies of Nutrients

The difference method and multivariate weighted–nested regression model were specifically used to estimate the energy value and energetic efficiency of individual nutrients (starch, soybean oil, and casein) from experimental diets in this study. The energetic efficiency of nutrients can significantly vary based on their origins and the metabolic pathways involved. van Milgen et al. [11] reported that the *kj* of starch in growing pigs was 0.84. In the current study, the *kj* of corn starch, pea starch, and tapioca starch fed to growing pigs were 0.88, 0.80, and 0.86, respectively. Notably, the energetic efficiency values of corn starch and tapioca starch was slightly greater than those from van Milgen et al. [11]. This discrepancy may stem from the exclusion of energy costs associated with digestion and metabolic processes from the calculations, such as glycogen reserves [11]. In contrast, the lower *kj* observed in pea starch can be attributed to the presence of amylose and resistant starches, which impede energy utilization, resulting in reduced energy efficiency [36]. The primary energy consumption of lipids occurs during the activation of fatty acids, with an energy loss of only 3% [11]. Consequently, from a theoretical perspective, the *kj* value of lipids should approach 97%. In this study, the *kj* in soybean oil was 0.95, aligning closely with the theoretical value. Meanwhile, this study determined the *pj* and *kj* values of casein to be 0.51 and 0.68, respectively, while van Milgen et al. [11] reported *pj* and *kj* values of 0.42 and 0.52 for casein, respectively. The relatively high *pj* and *kj* can be attributed to the accelerated protein metabolic turnover and consequent enhancement of ATP production from protein catabolism in animals consuming high-protein diets [37].

Starch exhibits significant variations in energy value due to its complex and diverse structure and composition, as well as the varying number of glycosidic bonds. In this study, the energy values of corn starch and pea starch were lower than those of tapioca starch. This could be attributed to the greater amylose content in corn and pea starches, which, compared to tapioca starch, has a lower efficiency in energy supply through fermentation in the hindgut, as opposed to the more readily degraded amylopectin that is converted to glucose in the foregut [38]. Additionally, glycosidic bonds in starch release energy during oxidative decomposition, which could also affect the available energy values of different kinds of starch [39]. Previous studies reported variations in the energy value of soybean oil, with DE values ranging from 35.9 to 38.1 MJ/kg, ME values ranging from 35.5 to 36.4 MJ/kg, and NE values ranging from 19.10 to 34.05 MJ/kg [12,27,40,41]. These values were similar to the DE, ME, and NE values of soybean oil evaluated in this study, which were 36.49 MJ/kg, 36.16 MJ/kg, and 34.03 MJ/kg, respectively. Our research determined that 30 kg pigs exhibited lower DE, ME, and NE than the values reported by van Milgen et al. [11] for 60 kg individuals, indicating a significant influence of body weight on the energy values of growing pigs. This difference could also be attributed to variations in the breed of pigs and the composition of basal diets. Specifically, van Milgen et al. [11] employed Pietrain × Landrace × Large White barrows, whereas the present study was based on Duroc × Landrace × Yorkshire barrows. Furthermore, the lower NE values in our study may be attributed to limitations of the difference method, which assumes no interactions between the basal diet and test nutrients; this assumption may introduce discrepancies [42]. In reality, the addition of casein in our experimental diets widens the gap in CP levels between basal and experimental diets, potentially interfering with protein digestion and resulting in NE underestimation of casein [43].

### 4.4. Correlation Analysis and Prediction Equations for PD and LD Based on Nutrient Characteristics of Different Ingredients

According to the factorial method, the NE partition in growing pigs is categorized into PD and LD, where nutrient variations in ingredients significantly impact the allocation ratio, a topic that has been insufficiently explored. In this study, the nutrient characteristics of ingredients were employed to investigate NE partition patterns. The correlation between nutrient characteristics and PD and LD values were consistent with previous findings. A significantly positive correlation exists between the CP level and PD (r = 0.92), while a negative correlation is observed with LD (r = −0.63), and the strongest correlations with LD are found with GE and EE at −0.94 and 0.93, respectively. This highlights the CP level as a crucial factor for PD and GE, and EE levels as determinants for LD. However, there was no significant correlation between starch content and PD and LD.

The coefficient of determination in a prediction equation, denoted as R^2^, signifies the degree to which the model accounts for variability in the dependent variable, with higher values suggesting a more precise fit [44]. Additionally, the lower RMSE, AIC, and BIC values suggest a smaller prediction error and enhanced model accuracy. Based on the above statistical metrics, a quadratic prediction equation was recommended as the best prediction equation for PD, which was PD = 364.36 − 18.44 × GE + 29.10 × CP − 3.79 × EE − 21.37 × ADF (R^2^ = 0.96; RMSE = 105.15), and a quintuple prediction equation was recommended as the best prediction equation for LD, which was LD = −1503.50 + 21.58 × CP + 51.98 × EE + 26.30 × Starch + 26.81 × NDF − 23.87 × ADF (R^2^ = 0.98; RMSE = 172.85). The current study firstly reported the effects of different nutrients on digestibility, energetic efficiency, and NE partition patterns in growing pigs, and developed prediction equations for PD and LD through multiple regression analysis based on the nutrient characteristics of ingredients. The approach developed in this study could offer novel insights and support for the advancement of precision swine nutrition.

## 5. Conclusions

There are considerable differences in energetic efficiency and NE partition patterns among various nutrients. Due to structural and compositional differences, corn starch and tapioca starch demonstrated greater energetic efficiencies than pea starch. Among different nutrients, the *kj* of soybean oil was 11% and 27% greater than that of starch and casein, respectively, while the *pj* of casein was 46% and 39% greater than starch and soybean oil, respectively. Finally, the NE values of corn starch, pea starch, tapioca starch, soybean oil, and casein in growing pigs were measured to be 15.06, 14.37, 15.22, 34.03, and 16.22 MJ/kg DM, respectively, and the optimal prediction equations for the PD and LD developed in this study were PD = 364.36 − 18.44 × GE + 29.10 × CP − 3.79 × EE − 21.37 × ADF (R^2^ = 0.96; RMSE = 105.15) and LD = −1503.50 + 21.58 × CP + 51.98 × EE + 26.30 × Starch + 26.81 × NDF − 23.87 × ADF (R^2^ = 0.98; RMSE = 172.85).

## Figures and Tables

**Table 1:** Ingredient composition and nutrient levels of the experimental diets (%, as-fed basis).

Items	Diets					
	T1	T2	T3	T4	T5	T6
Ingredient, %						
Corn	72.90	51.44	51.44	51.44	68.75	64.39
Soybean meal	23.20	16.37	16.37	16.37	21.88	20.49
Corn starch	-	27.00	-	-	-	-
Pea starch	-	-	27.00	-	-	-
Tapioca starch	-	-	-	27.00	-	-
Soybean oil	-	-	-	-	5.00	-
Casein	-	-	-	-	-	11.80
Dicalcium phosphate	0.85	1.00	1.00	1.00	0.90	0.80
L-lysine-HCl	0.50	0.96	0.96	0.96	0.73	-
DL-methionine	0.09	0.23	0.23	0.23	0.12	0.05
L-threonine	0.10	0.25	0.25	0.25	0.13	0.01
L-tryptophan	0.04	0.08	0.08	0.08	0.05	0.04
L-valine	0.01	0.21	0.21	0.21	0.05	0.01
Salt	0.21	0.26	0.26	0.26	0.29	0.31
Limestone	1.10	1.20	1.20	1.20	1.10	1.10
Premix ^1^	1.00	1.00	1.00	1.00	1.00	1.00
Total	100.00	100.00	100.00	100.00	100.00	100.00
Nutrient levels ^2^						
DM	85.99	85.91	86.23	85.83	87.74	85.09
Ash	4.36	4.18	4.23	4.13	4.41	4.23
EE	2.90	2.05	2.01	2.03	6.92	2.50
NDF	11.14	7.83	7.40	7.28	9.81	9.67
ADF	4.03	2.70	2.29	2.47	3.27	3.07
CP	16.31	12.35	12.08	12.19	16.03	23.58
GE, MJ/kg	17.53	17.09	17.15	17.22	18.33	17.84
Lys/ME, g/Mcal	2.41	2.39	2.42	2.39	2.48	3.41
Lysine	0.99	1.01	1.01	1.01	1.07	1.44
Methionine + cysteine	0.58	0.57	0.57	0.57	0.58	0.82
Threonine	0.61	0.60	0.60	0.60	0.61	0.87
Tryptophan	0.18	0.17	0.17	0.17	0.18	0.31
Valine	0.67	0.67	0.67	0.67	0.67	1.25

^1^ The premix provided the following per kilogram of the diet: vitamin A, 12,500 IU; vitamin D_3_, 1500 IU; vitamin E, 15 IU; vitamin K_3_, 2.0 mg; thiamine, 1.0 mg; riboflavin, 3.0 mg; pyridoxine, 1.5 mg; VB_12_, 0.01 mg; pantothenic acid, 15 mg; nicotinic acid, 30 mg; biotin, 0.05 mg; folic acid, 1.5 mg; Zn, 70 mg; Fe, 55 mg; Mn, 12 mg; Cu, 10 mg; I, 0.5 mg; Se, 0.4 mg. ^2^ The lysine, methionine + cysteine, threonine, tryptophan, and valine were calculated values, and the rests were analyzed values. DM, dry matter; EE, ether extract; NDF, neutral detergent fiber; ADF, acid detergent fiber; CP, crude protein; GE, gross energy.

**Table 2:** The variables used in Equations (8)–(12), including their definitions and units.

Variables	Definitions	Units
NE_i_	net energy of experimental diet	MJ/kg DM
ME_i_	metabolizable energy of experimental diet	MJ/kg DM
HI	heat increment	kJ/kg BW^0.6^/d
RE_i_	retention energy of experimental diet	kJ/kg BW^0.6^/d
THP	total heat production	kJ/kg BW^0.6^/d
PD_i_	protein deposition of experimental diet	kJ/kg BW^0.6^/d
PD-free NE_i_	net energy not deposited as protein of experimental diet	kJ/kg BW^0.6^/d
LD_i_	lipid deposition of experimental diet	kJ/kg BW^0.6^/d

**Table 3:** The variables used in Equations (13)–(18), including their definitions and units.

Variables	Definitions	Units
energy value_j_	energy value of nutrients	MJ/kg DM
energy value_test_	energy value of test diet (T2–T6 diets)	MJ/kg DM
energy value_basal_	energy value of basal diet (T1 diet)	MJ/kg DM
r_0_	the energy-supplying fractions (consisting of corn and soybean meal) in the basal diet	%
r_1_	the proportion of the basal ingredients in the test diet	%
r_2_	the proportion of the tested nutrients in the test diet	%
GE_j_	gross energy of nutrients	MJ/kg DM
DE_j_	digestible energy of nutrients	MJ/kg DM
*dj*	efficiency of using GE for DE of nutrients	-
ME_j_	metabolizable energy of nutrients	MJ/kg DM
*mj*	efficiency of using DE for ME of nutrients	-
PD_j_	protein deposition of nutrients	kJ/kg BW^0.6^/d
*pj*	efficiency of using ME for PD of nutrients	-
PD-free NE_j_	net energy not deposited as protein of nutrients	kJ/kg BW^0.6^/d
*kj*	efficiency of using ME for PD-free NE of nutrients	-
LD_j_	lipid deposition of nutrients	kJ/kg BW^0.6^/d
K_BR_	the efficiency of using body reserves for ATP synthesis	-
FHP	fasting heat production	kJ/kg BW^0.6^/d
NE_PD_	the additional energy required for PD	-

**Table 4:** The nutrient information of different ingredients evaluated in our previous studies ^1^.

Ingredients	Nutrient Characteristics (%)								
	GE, MJ/kg	CP	EE	Starch	NDF	ADF	Ash	PD, kJ/kg BW^0.6^/d	LD, kJ/kg BW^0.6^/d
Peanut meal	19.17	52.63	1.90	15.23	18.87	7.43	6.85	1339.02	456.01
Soybean meal	19.17	52.97	0.88	3.50	15.00	6.68	7.07	1385.60	7.60
Soybean meal	19.47	51.23	2.10	3.50	17.55	7.76	6.41	1361.60	5.95
Soybean meal	19.29	50.25	1.28	3.50	19.73	9.17	6.56	1277.60	3.60
Soybean meal	19.12	49.25	0.93	3.50	17.46	7.40	6.83	1337.60	35.60
Soybean meal	19.50	48.52	1.20	0.00	17.08	9.96	6.21	1195.06	5.59
Soybean meal	19.21	48.02	1.25	3.50	15.73	7.74	6.27	1321.60	15.60
Cottonseed meal	17.99	46.43	0.27	1.80	33.02	15.35	6.28	1098.74	31.13
Rapeseed meal	19.54	42.36	1.08	0.00	30.75	20.7	6.97	1530.06	115.59
Rapeseed meal	19.37	41.83	1.68	2.26	39.83	24.79	9.14	730.25	98.34
Rapeseed meal	19.60	41.03	1.71	3.12	30.84	20.04	7.22	694.35	75.26
Rapeseed meal	19.47	39.92	2.58	3.02	37.27	21.50	8.24	637.94	98.34
Rapeseed cake	20.56	39.75	6.55	4.43	32.34	21.75	6.94	689.22	31.67
Rapeseed cake	21.33	39.23	9.52	0.00	36.98	22.84	6.41	815.06	0.00
Rapeseed meal	18.11	38.23	1.02	3.58	25.88	15.64	6.54	750.02	26.01
Rapeseed cake	21.35	37.70	11.27	2.95	41.00	24.18	7.49	196.91	642.69
Sunflower meal	19.13	32.49	1.93	4.91	43.51	28.97	8.51	475.60	19.34
DDGS	20.16	26.95	10.58	3.97	39.93	12.23	4.99	406.98	706.00
Corn gluten feed	18.58	23.05	2.34	14.85	42.27	12.70	5.70	437.40	241.23
Corn germ meal	19.31	21.53	2.07	19.54	50.30	14.38	1.85	342.26	453.53
Full-fat rice bran	20.61	15.30	16.04	32.92	17.78	7.38	8.11	343.12	809.09
Corn	18.84	8.71	4.06	74.19	10.22	1.35	1.51	193.08	1011.87
Corn	18.79	8.77	3.74	74.19	10.85	2.32	1.67	206.40	904.02
Corn	18.53	8.61	3.68	74.28	9.82	1.07	2.05	181.71	1006.61
Corn	18.76	8.54	3.75	74.64	10.36	2.08	1.77	182.66	1002.06
Corn	18.71	9.51	3.86	71.93	10.94	2.10	1.7	233.37	1016.11
Corn	18.74	9.34	3.62	71.90	10.93	1.96	1.47	195.13	1027.26
Corn	16.37	7.60	3.32	66.44	13.06	2.83	1.13	197.74	965.94
Corn	16.02	8.31	2.16	61.03	7.21	1.58	1.06	112.75	76.43
Corn	16.02	8.31	2.16	61.03	7.21	1.58	1.06	112.16	311.06
Corn	16.02	8.31	2.16	61.03	7.21	1.58	1.06	360.91	540.67
Wheat	16.48	13.76	1.59	60.78	12.38	3.15	1.67	350.26	761.66
Wheat	16.19	14.51	1.76	50.85	13.30	2.31	1.78	367.67	712.84
Husked millet	17.11	14.27	1.23	65.16	8.29	1.56	1.21	410.03	970.96
Millet	17.49	12.17	1.88	51.34	22.74	11.07	2.34	330.46	733.66
Naked oat	17.06	15.31	3.68	59.30	12.73	3.14	1.83	398.22	796.43
Sorghum	16.42	10.13	2.79	61.66	11.37	3.78	1.55	210.80	828.66
Barley	16.21	9.68	1.63	54.43	24.68	6.62	2.38	276.23	806.95
Unpolished rice	15.88	9.99	1.56	70.81	10.26	1.68	1.15	276.45	974.32
Partially husked barley	16.33	9.95	1.89	58.35	20.78	2.98	1.67	281.87	895.34
Soybean oil	0.00	0.35	93.65	0.00	0.00	0.00	3.24	8.12	2823.49
Soybean oil	0.00	0.35	94.65	0.00	1.13	0.00	0.00	65.08	3728.48
Poultry oil	0.00	0.00	91.40	0.00	0.00	0.00	3.20	6.77	3008.14
Linseed oil	0.00	0.00	91.00	0.00	0.00	0.00	2.70	7.66	3208.03
Fish oil	0.00	0.00	86.90	0.00	0.00	0.00	2.10	86.77	3158.47
Corn oil	0.00	0.00	88.30	0.00	0.00	0.00	3.20	6.77	3208.05
Palm oil	0.00	0.00	91.50	0.00	0.00	0.00	4.00	66.77	3158.11

^1^ The data are presented as mean values, with GE, CP, EE, starch, NDF, ADF, and ash analyzed in duplicate, while other parameters were determined in six replicates. GE, gross energy; CP, crude protein; EE, ether extract; NDF, neutral detergent fiber; ADF, acid detergent fiber; DDGS, corn distillers dried grains with soluble.

**Table 5:** Effects of different nutrients on apparent total tract digestibility of nutrients and nitrogen balance when fed to growing pigs.

Items	Diets						SEM	*p*-Value
	T1	T2	T3	T4	T5	T6		
BW, kg	30.66	30.63	29.69	29.62	30.64	30.72	0.28	0.76
DM intake, g/d	935	897	884	878	874	889	21.28	0.95
Apparent digestibility/%								
GE	87.28 ^b^	91.23 ^a^	90.97 ^a^	91.59 ^a^	88.89 ^ab^	88.98 ^ab^	0.38	<0.01
OM	87.19 ^b^	91.51 ^a^	91.27 ^a^	91.82 ^a^	88.91 ^ab^	88.96 ^ab^	0.41	<0.01
CP	86.90	88.68	87.74	89.26	86.26	90.01	0.47	0.16
EE	54.75 ^b^	54.60 ^b^	53.70 ^b^	50.39 ^b^	79.47 ^a^	52.60 ^b^	2.11	<0.01
NDF	54.87	56.85	55.60	54.62	56.15	50.91	1.11	0.74
ADF	48.13	47.90	43.94	51.83	45.99	39.22	1.45	0.20
Nitrogen balance/(g/d)								
Nitrogen intake	28.38 ^b^	20.63 ^d^	19.83 ^d^	19.96 ^d^	25.57 ^c^	39.39 ^a^	1.30	<0.01
Fecal nitrogen	3.71 ^a^	2.32 ^b^	2.43 ^b^	2.13 ^b^	3.52 ^a^	3.94 ^a^	0.16	<0.01
Urinal nitrogen	4.44 ^b^	3.69 ^b^	3.12 ^b^	3.43 ^b^	3.68 ^b^	8.34 ^a^	0.38	<0.01
Nitrogen retention	20.22 ^b^	14.62 ^c^	14.27 ^c^	14.40 ^c^	18.37 ^b^	27.11 ^a^	0.89	<0.01
Net protein availability, %	0.71 ^a^	0.71 ^a^	0.72 ^a^	0.72 ^a^	0.72 ^a^	0.69 ^b^	0.02	<0.01

Means within a row with different letters differ among different dietary treatments (*p* < 0.01), *n* = 6. BW, body weight; DM intake, dry matter intake; GE, gross energy; OM, organic matter; CP, crude protein; NDF, neutral detergent fiber; ADF, acid detergent fiber.

**Table 6:** Effects of different nutrients on energy balance and utilization when fed to growing pigs.

Items	Diets						SEM	*p*-Value
	T1	T2	T3	T4	T5	T6		
Energy balance, kJ/kg BW^0.6^/d								
MEI	2066.73	2036.18	2018.06	2039.59	2027.80	2013.16	30.53	0.64
THP	1176.64	1150.69	1173.54	1168.28	1093.23	1112.11	39.03	0.06
FHP	853.09	854.36	827.46	823.37	834.21	847.62	48.16	0.96
Total RE	890.09	885.49	844.52	871.31	934.57	901.05	53.13	0.54
PD	386.42^b^	279.48^c^	278.27^c^	281.00^c^	351.86^b^	518.03^a^	16.57	<0.01
LD	503.66^a^	606.01^a^	566.25^a^	590.30^a^	582.71^a^	383.02^b^	17.44	<0.01
RQ	1.03	1.03	1.03	1.04	1.01	1.01	-	-
Fasted RQ	0.83	0.81	0.81	0.81	0.82	0.83	-	-
Energy values, MJ/kg DM								
DE	17.79^b^	18.15^ab^	18.09^ab^	18.38^ab^	18.57^a^	18.66^a^	0.07	<0.01
ME	17.23^b^	17.68^ab^	17.45^b^	17.72^ab^	18.07^a^	17.69^ab^	0.07	<0.01
NE	14.50^b^	15.13^ab^	14.49^b^	14.78^ab^	15.76^a^	15.43^ab^	0.13	<0.01
Energy utilization, %								
ME/DE	97.00^a^	97.20^a^	96.60^a^	96.40^a^	97.00^a^	94.80^b^	0.74	<0.01
NE/ME	84.20	85.60	83.00	83.40	87.00	87.40	2.31	0.11

Means within a row with different letters differ among different dietary treatments (*p* < 0.01), n = 6. MEI, metabolizable energy intake; THP, total heat production; FHP, fasting heat production; RE, retention energy; PD, protein deposition; LD, lipid deposition; RQ, respiratory quotient; GE, gross energy; DE, digestible energy; ME, metabolizable energy; NE, net energy.

**Table 7:** The energy value and energetic efficiency of different nutrients fed to growing pigs.

Items	Basal	Corn Starch	Pea Starch	Tapioca Starch	Soybean Oil	Casein	SEM
Utilization efficiency of energy							
*dj*	0.87	0.99	0.99	1.00	0.96	0.95	0.01
*mj*	0.97	0.99	0.95	0.95	0.99	0.88	0.01
*pj*	0.19	0.05	0.05	0.05	0.12	0.51	0.03
*kj*	0.81	0.88	0.80	0.86	0.95	0.68	0.02
NE_PD_	0.35						
K_BR_	0.96						
Energy values, MJ/kg DM							
GE	18.36	16.13	16.30	17.02	37.97	20.20	1.43
DE	16.02	16.03	16.06	17.07	36.49	19.18	1.39
ME	15.52	15.77	15.37	16.34	36.16	16.89	1.42
NE	14.50	15.06	14.37	15.22	34.03	16.22	1.80

*dj*, efficiency of using GE for DE; *mj*, efficiency of using DE for ME; *pj*, efficiency of using ME for PD; *kj*, efficiency of using ME for PD-free NE; NE_PD_, additional energy required for PD; K_BR_, efficiency of using body reserves for ATP synthesis; GE, gross energy; DE, digestible energy; ME, metabolizable energy; NE, net energy.

**Table 8:** Prediction equations of protein deposition and lipid deposition from nutrient characteristics of ingredients fed to growing pigs.

Multiple Regression Equations	R^2^	RMSE	AIC	BIC	Validation R^2^
PD = −23.37 + 23.50 × CP	0.87	173.36	373.09	376.08	0.78
PD = 37.56 + 28.98 × CP − 24.51 × ADF	0.96	105.45	346.89	350.48	0.77
PD = 48.64 + 29.64 × CP − 23.77 × ADF − 7.77 × Ash	0.96	106.99	349.54	353.48	0.77
PD = 364.36 − 18.44 × GE + 29.10 × CP − 3.79 × EE − 21.37 × ADF	0.96	105.15	350.66	354.65	0.80
PD = 509.27 − 17.47 × GE + 26.61 × CP − 5.41 × EE − 2.01 × Starch − 24.37 × ADF	0.96	106.18	353.56	357.28	0.79
PD = 640.63 − 17.12 × GE + 24.56 × CP − 6.88 × EE − 3.36 × Starch − 2.49 × NDF − 21.91 × ADF	0.96	108.06	357.21	360.29	0.80
PD = 643.11 − 18.60 × GE + 24.54 × CP − 7.02 × EE − 3.13 × Starch − 2.21 × NDF − 22.21 × ADF + 3.45 × Ash	0.96	110.66	361.60	363.59	0.80
LD = 3106.14 − 141.09 × GE	0.88	359.68	413.96	416.95	0.88
LD = 1.25 + 35.15 × EE + 11.46 × Starch	0.96	199.62	382.62	386.21	0.97
LD = −200.77 + 37.34 × EE + 13.10 × Starch + 6.37 × NDF	0.97	193.09	382.61	386.54	0.97
LD = −1276.12 + 16.77 × CP + 49.36 × EE + 24.81 × Starch + 15.65 × NDF	0.97	183.93	381.97	385.96	0.97
LD = −1503.50 + 21.58 × CP + 51.98 × EE + 26.30 × Starch + 26.81 × NDF − 23.87 × ADF	0.98	172.85	380.85	384.57	0.97
LD = −1424.08 − 5.51 × GE + 21.91 × CP + 51.11 × EE + 26.46 × Starch + 26.91 × NDF − 22.82 × ADF	0.98	176.67	384.74	387.82	0.97
LD = −1413.85 − 11.61 × GE + 21.86 × CP + 50.55 × EE + 27.41 × Starch + 28.06 × NDF − 24.06 × ADF + 14.23 × Ash	0.98	180.37	388.96	390.95	0.97

PD, protein deposition; LD, lipid deposition; GE, gross energy; CP, crude protein; EE, ether extract; NDF, neutral detergent fiber; ADF, acid detergent fiber; RMSE, root mean square error; AIC, Akaike Information Criterion; BIC, Bayesian Information Criterion.

## Data Availability

The data that support the study findings are available from the first author upon request.

## Abbreviations

The following abbreviations are used in this manuscript:

ADF	Acid detergent fiber
AIC	Akaike Information Criterion
ATTD	Apparent total tract digestibility
BIC	Bayesian Information Criterion
BW	Body weight
CH_4_E	Methane energy
CP	Crude protein
DE	Digestible energy
d_j_	Efficiency of using GE for DE
DDGS	Corn distillers dried grains with soluble
DM	Dry matter
DM intake	Dry matter intake
EE	Ether extract
FE	Fecal energy
FHP	Fasting heat production
GE	Gross energy
HI	Heat increment
HP	Heat production
K_BR_	Efficiency of using body reserves for ATP synthesis
*kj*	Efficiency of using ME for PD-free NE
LD	Lipid deposition
ME	Metabolizable energy
MEI	Metabolizable energy intake
ME_m_	Metabolizable energy for maintenance
m_j_	Efficiency of using DE for ME
NDF	Neutral detergent fiber
NE	Net energy
NE_PD_	Additional energy required for PD
OM	Organic matter
PD	Protein deposition
PD-free NE	Net energy not deposited as protein
*pj*	Efficiency of using ME for PD
RE	Retention energy
RMSE	Root mean square error
RQ	Respiratory quotient
THP	Total heat production
UE	Urine energy
